# Evaluating a Low-Cost Technology to Enable People with Intellectual Disability or Psychiatric Disorders to Initiate and Perform Functional Daily Activities

**DOI:** 10.3390/ijerph18189659

**Published:** 2021-09-14

**Authors:** Emanuela Resta, Lucia Brunone, Fiora D’Amico, Lorenzo Desideri

**Affiliations:** 1 Department of Medical and Surgical Sciences, University of Foggia, 71122 Foggia, Italy; restaemanuela@gmail.com; 2 Psychiatric Rehabilitation Service “Incontri”, 70017 Bari, Italy; lucia.brunone@libero.it; 3 Silver House Health and Care Services, 70011 Bari, Italy; damicofiora87@gmail.com; 4 Department of Psychology, University of Bologna, 40126 Bologna, Italy

**Keywords:** assistive technology, low-cost solutions, functional activities, independence, intellectual disability

## Abstract

People with intellectual disability or psychiatric disorders and cognitive dysfunctions may need assistive technology to maintain and improve their levels of functioning and independence. This study assessed a smartphone-based system to remind the user to initiate functional daily activities (e.g., setting a table for lunch) and perform them without the support of a caregiver. The system was evaluated through a non-concurrent multiple baseline design across two groups of participants. During the intervention sessions, the participants were provided with a system involving a Samsung Galaxy A3 smartphone fitted with the Easy Alarm YouTube application and audio files. The alarm served to remind the participant to carry out a planned activity. Following the reminder, the smartphone presented each of the step instructions preset for the activity. The data showed a statistically significant increase in the number of activities initiated independently from baseline to intervention for all participants. All participants also showed a significant increase in the number of activity steps correctly performed when supported by the smartphone. These results suggest that a low-cost system (i.e., smartphone) can be used to improve independence of people with intellectual disability or psychiatric disorders with cognitive dysfunctions.

## 1. Introduction

People with intellectual disability or psychiatric conditions and cognitive dysfunctions may have serious difficulties in managing functional daily activities [1,2,3,4]. In fact, they may fail to start the activities independently and wait for reminders from staff or caregivers, or may fail to start them at the appropriate time. Moreover, they may not remember part or many of the steps involved in the activities and/or the correct sequence of those steps [4,5,6]. As a consequence of this situation, they may remain largely dependent on external cues and guidance from others (i.e., parents or caregivers) with negative implications for their self-confidence, social image, constructive occupation time [7], as well as social costs [8].

Large consensus exists on the need to address such situation by helping the people to reduce their level of dependence and develop a more active and functional role within their daily context [9,10,11]. Efforts in this direction have been increasingly based on the use of low-cost (affordable) commercially available mainstream technology devices, such as tablets and smartphones [12,13,14]. These technology devices can be programmed to present simple pictorial instructions, brief video clips (video prompts), verbal instructions, or combinations of visual and verbal instruction forms [15]. Compared to traditional assistive technologies specifically developed for people with disabilities, mainstream mobile technology devices have the advantage of being more accessible in that they can be easily customized according to the user’s characteristics and preferences [16], as well as easier to transport from one setting to another [13,17] and more socially accepted [18].

According to the findings of a literature review in the area [19], in many studies, the participant was required to self-operate the device available (e.g., touch an icon on the device screen) in order to get each of the instructions for the activities programmed (e.g., to make a sandwich [20]). After managing to get an instruction, the participant was to carry out the activity step represented by that instruction; then, the participant was expected to operate the device again to get the following instruction and carry out the related step. The process was repeated the same way for each of the remaining instructions and activity steps until the entire sequence was ended [19]. 

In other studies, the participant was not required to operate the device to get the instructions (e.g., [21,22,23,24]) in that the instructions were automatically presented by the device at preset time intervals. The length of the interval between each given instruction (i.e., separating one instruction from the next) was decided by a member of the staff or a caregiver based on his/her knowledge/expectation of the time needed for the participant to carry out the instruction-related step. 

The results of both groups of studies were largely encouraging, showing that the use of technology to present instructions for the single steps of the activities to be carried out can have a positive impact on the participants’ performance (as shown in [19]). Notwithstanding the overall apparent success of the technology in guiding the participants’ activity performance, two issues may require to be addressed. First, very limited attention was focused on extending the use of the technology to also include the participants’ independent start of the activities at the appropriate time. Yet, this aspect would seem to be relevant and could be profitably included in a technology-aided intervention program. Second, the use of technology has been mostly limited to no more than three activities [19]. Yet, one could see the technology being employed for a variety of activities spread over the day so as to provide a practically relevant support to the participants’ initiative and engagement.

In light of these considerations, the present exploratory study was aimed at answering the following research question: can adults diagnosed with mild to moderate intellectual disability or psychiatric disorders with cognitive dysfunctions use a commercially available smartphone to independently initiate and perform functional daily activities? To answer this question, we provided 14 adults with a smartphone that alerted them as to the time when any particular activity scheduled for the day was to be started and then provided with the verbal instructions for the single steps of that activity. The instructions were automatically presented by the smartphone at preset intervals. This instruction presentation strategy was thought to be advantageous compared to a strategy requiring the participant to provide an input (e.g., touch an icon) to receive any new instruction. In fact, the automatic presentation frees the participant from the burden of remembering to seek every single instruction and of possessing motor skills adequate for activating icons or scrolling on a smartphone screen [19].

The current study may be relevant given the increasing need to (a) help caregivers and support staff to improve the effectiveness of their interventions [25], and (b) provide people with intellectual disability [26,27] or psychiatric disorders [28] with affordable solutions to achieve independence in daily activities. 

## 2. Materials and Methods

### 2.1. Participants

The participants were recruited for the study on the basis of specific criteria that have been verified through preliminary observations and staff interviews. First, they were interested in performing daily functional activities, but generally, they needed support to carrying them out. Second, they could carry out simple functional daily activities if provided with verbal guidance. Third, they had expressed the interest in using smartphones that would guide them to perform a variety of daily activities. Fourth, the professional caregivers/staff had expressed their full support for the technology-based program here reported, which had been shown to them in advance. 

Based on these criteria, the study involved a convenience sample of 14 participants that were divided into two groups based on their primary/main diagnosis [29]. Specifically, Group 1 involved participants with psychiatric conditions with comorbid cognitive impairments. Group 2 included participants whose main diagnosis involved intellectual disability. 

Table 1 lists the participants by their pseudonyms and report their chronological ages and their primary/main diagnoses. Other information reported in the tables include (a) the overall intellectual functioning based on the Italian version of the mini-mental state examination (MMSE), which was scored according to the normative data reported in [30]; (b) the participants’ capabilities related to executive functioning and cognitive control assessed by means of the frontal assessment battery (FAB) [31,32]; and (c) the degree to which the participants could perform basic activities of daily living (ADL), such as bathing or dressing [33], as well as more complex instrumental activities of daily living (IADL), such as doing laundry or handling finances [34]. 

Group 1 included eight participants (four females). Their chronological ages ranged from 45 to 62. Seven participants presented with a main diagnosis of schizophrenia or schizoaffective disorder and one participant presented with major depressive disorder. All the participants in the first group presented with a global impairment of the intellectual and the executive functioning, that is, all the participants’ MMSE and FAB scores resulted below the cut-off point of 24 and 2, respectively [30,32]. Moreover, all participants in this group had problems in completing some basic as well as instrumental activities of daily living as reported in Table 1 (see columns labeled ADL and IADL, respectively). 

Group 2 included six participants (two females). Their chronological ages ranged from 25 to 40. All participants in this group presented with a main diagnosis of intellectual disability. Similar to those in the first group, all the participants in the second group reported MMSE and FAB scores below the cut-off points, indicating marked intellectual as well executive functioning difficulties. In addition, none of the participants involved in the second group could independently complete all the basic (instrumental) activities of daily living assessed.

The participants’ interest in using the technology system was taken as consent for the study. However, given the participants’ inability to read and sign a consent form, their legal representatives were asked to sign such a form on their behalf. All legal representatives signed the consent form. The study complied with the 1964 Helsinki declaration and its later amendments and was approved by an institutional Ethics Committee.

### 2.2. Settings, Sessions and Activities

The participants attended two different rehabilitation and care facilities aimed at providing them with education/rehabilitation and occupation opportunities. These centers served as the setting for carrying out the activities during the baseline and intervention phases of the study. The activities consisted of daily tasks concerning self-help, domestic, and occupational forms of engagement. The activities were typically selected and set up for the individual participants; that is, different activities could be available for different participants. Moreover, the number and types of steps involved in activities available for different participants could vary across them. Per day, 8 to 14 (with a mean (*M*) of about 10) activities were planned. Those activities could involve the morning bathroom routine, dressing, dental hygiene, breakfast routine, food preparation, cooking classes (each of which consisted of making a specific type of food), exercise, preparing to go home, cleaning the room, working in the garden, and operating a PC to see slides or book material. The number of steps included in those activities varied between 12 and 21 (*M* = 15). Table 2 provides a list of steps (i.e., and the possible verbal instructions the participants received) for two of those activities.

### 2.3. Measures and Recording

During the first baseline, the measure recorded consisted of the number of activities scheduled for the day that the participants started independently. During the second baseline and the intervention, the measures recorded concerned the number of activities started independently as well as the number of activity steps carried out independently. Recording was carried out by staff responsible for the participants’ daily programs. Reliability observers (other staff or research personnel) were also employed to check interrater agreement. Those observers independently recorded between 20% and 30% of the data collected for each participant (i.e., activities started independently, and activity steps carried out correctly/independently). The percentage of interrater agreement was computed for each participant by dividing the number of agreements by total number of agreements plus disagreements and multiplying by 100%. The agreements were the number of activities started independently and the number of activity steps on which regular staff and the reliability observer had the same score, that is, “independent” or “dependent” (for the activities started) and “correct” or “incorrect” (for the activity steps). Overall, high rates of agreement between the regular staff and the reliability observer were observed, with percentages of interrater agreement in the 80–100 ranges and average agreement scores above 92% for each measure of each participant.

#### 2.3.1. Technology System

The technology available to each participant during the intervention phase of the study was a Samsung Galaxy A3 smartphone with Android 9 Operating System (Mountain View, CA, USA). The smartphone included standard functions, such as Bluetooth connection and an alarm. For the scopes of the present study, it was fitted with the Easy Alarm YouTube application and included audio files presenting the verbal reminders for the start of the activities and the instructions concerning the activity steps. For each of the activities available, one audio file was used. The staff responsible for the participants’ daily planning was in charge of setting the time for the performance of each of the activities scheduled for that day. For each activity, a specific audio file presenting the instructions was linked to the alarm of the smartphone. At the time when the activity was scheduled for the day, the smartphone emitted a verbal reminder asking the participant to carry out such an activity. Following the reminder, which could be repeated, the smartphone presented each of the step instructions preset for the activity. The regular staff, who knew the participants, scheduled the time gaps/intervals between the reminder and the first activity instruction as well as between any pair of all the other instructions of the sequence, based on their knowledge of the participants and specific observations of their performance speed. Time intervals between activity-related instructions could change according to participants’ characteristics and needs. For instance, longer time intervals were set after instructions when the participant was requested to perform more demanding steps. The intervals could be readjusted over sessions according to the participant’s progress. The participants could listen to the instructions delivered by the smartphone through a wireless Bluetooth earpiece. In that way, the participants were not requested to carry the smartphone during the sessions.

#### 2.3.2. Experimental Conditions and Data Analysis

The study was conducted according to a non-concurrent multiple baseline design across participants [35]. The two groups were exposed to the same experimental design. Prior to the start of the intervention with the smartphone, two baseline phases were conducted. Each baseline phase included different numbers of sessions for the different participants of the groups. The intervention sessions were aimed at determining the effects of the smartphone on each of the two measures. The baseline and intervention daily percentages of activities started correctly, and activity steps carried out correctly were summarized/graphed as means per session over blocks of sessions. The differences between the two baselines and intervention session data on the single measures of each participant were further analyzed via the Kolmogorov–Smirnov test [36].

#### 2.3.3. Baseline I

The aim of baseline I was to assess whether the participants could start the activities correctly without the support of the smartphone and included two to five sessions. At the beginning of the day, staff provided the participants with a list of the activities that they were to carry out during the day and the times at which the activities were due.

#### 2.3.4. Baseline II

Baseline II served to assess the activity steps that the participants performed correctly. The participant did not have the smartphone nor the list. Specifically, staff asked the participants to carry out the activities scheduled for them (i.e., one at a time) to determine the number of steps the participants could carry out correctly (i.e., matching the task-analysis step specification). Staff provided verbal or physical prompting only if the participants made a mistake that precluded any continuation.

#### 2.3.5. Intervention

The intervention phase was aimed at assessing the effects of the smartphone on the participants’ independent/timely start of the planned activities as well as the correct performance of the activity steps. The intervention was carried out over 29–48 days. The participants had the smartphone, which was set up to provide verbal reminders at the time an activity was due and then the verbal instructions for the single activity steps (i.e., as described in the Technology system section). Prior to the start of the intervention phase, staff guided the participants through familiarization with the technology, reminders, and activity instructions over two days, during which the participants received verbal or physical prompting to familiarize/practice with the smartphone’s reminders as well as with the activity instructions delivered via the audio files. During the intervention phase that followed the familiarization/practice days, staff provided the participants with the smartphone set up for the activities scheduled during that day. Staff intervened with prompting/support only if the participants made step errors that would interfere with the continuation of the planned activity. The participants were generally praised by staff after the performance of the activities.

## 3. Results

**Figure 1:**
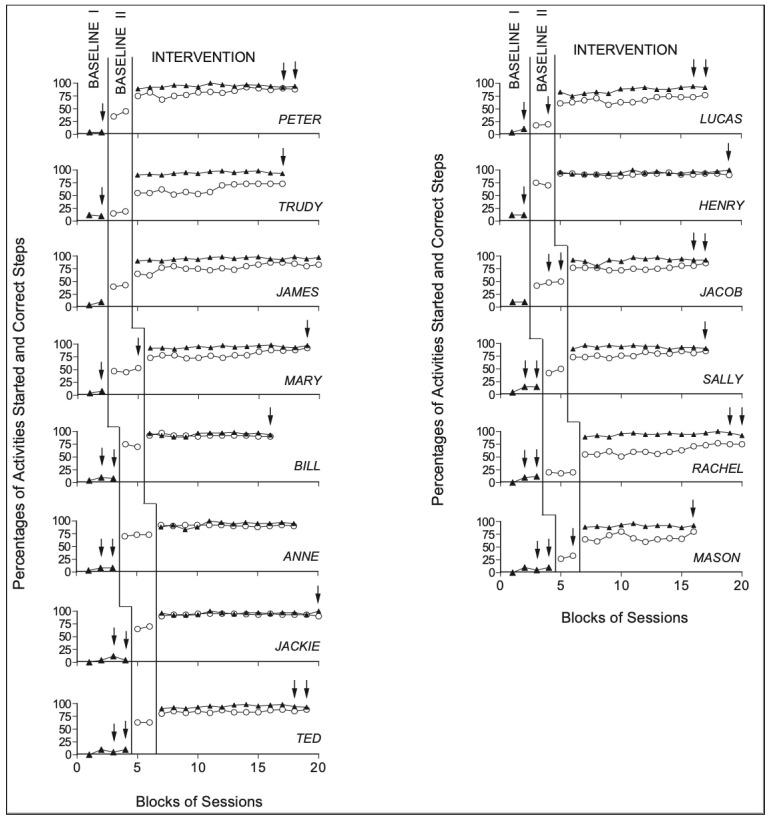
The eight panels to the left of the figure summarize the baseline and intervention data of the eight participants of Group 1 (i.e., Peter, Trudy, James, Mary, Bill, Anne, Jackie, and Ted). The six panels to the right of the figure summarize the baseline and intervention data of the six participants of Group 2 (i.e., Lucas, Henry, Jacob, Sally, Rachel, and Mason). The black triangles and empty circles represent mean percentages of activities started independently and of activity steps carried out correctly per day over blocks of days, respectively. The blocks include 3 days (2 days when an arrow is present).

Figure 1 reports the baseline and intervention data for the eight participants of Group 1 (i.e., the eight panels to the left of the figure) and the six participants of Group 2 (i.e., the six panels to the right of the figure). The black triangles and empty circles represent mean percentages of activities started independently and of activity steps carried out correctly per day over blocks of days, respectively. The blocks include 3 days (2 days when an arrow is present).

The data of Group 1 show that (a) during baseline I, the mean percentage of activities started independently per day was below 20% for each of the eight participants, while (b) during baseline II, the mean percentage of activity steps carried out correctly per day varied between below 20% and approximately 75%. During the intervention phase, the results show a marked increase in the mean percentage of activities started independently per day compared to baseline I, with percentages above 90% for all participants. The positive impact of the intervention was also evident for the number of activity steps carried out correctly per day, which varied between about 65% and over 90%. The Kolmogorov–Smirnov test further showed that the differences between the baseline data and the intervention data (i.e., single-day data points) on activities started independently and activity steps carried out correctly were statistically significant (*p* < 0.01) for all participants in Group 1.

The data of Group 2 were similar to those of Group 1, further indicating the positive impact of the intervention in enabling participants to both initiate and perform functional activities. Indeed, the percentage of activities started independently was below 20% for all participants (see baseline 1). In baseline II, the percentage of activity steps carried out correctly ranged from about 20% to approximately 70%. During the intervention, a marked increase in the number of activities started independently was observed, with percentages near and above 90% across participants. With regard to activity steps carried out correctly, also for participants in Group 2, a marked increase compared to baseline II was observed, with percentages ranging between approximately 65% and 85%. Importantly, the differences between baseline and intervention were statistically significant (*p* < 0.01) on both measures for all participants in Group 2.

## 4. Discussion

This study was aimed at evaluating the use of a low-cost solution to remind persons with intellectual disability or psychiatric disorders and cognitive dysfunctions to start functional/occupational activities and perform them independently. The results show that, in the intervention phase, all participants were successful in starting the activities as well as carrying them out with high levels of accuracy. The data also showed that most (85%) of the participants achieved high levels of accuracy in the number of steps performed correctly. Overall, the results extend previous evidence [19] and confirm that a simple and affordable mobile device can be programmed by caregivers and used by people with either intellectual disability or psychiatric disorders to achieve independence in activities of daily life. In light of these results, some considerations may be put forward.

First, the data of this study represent a new demonstration that it is possible to support the daily activity engagement of people with intellectual disabilities and/or psychiatric disorders through very simple technology, thus adding to the previous evidence in the field [21,22,23,24]. The new aspect of these data is that (a) the number of activities programmed during the day was rather large (larger than that reported by previous studies [19]), and (b) the participants were not only assisted in carrying out the activities but also at starting them at the appropriate time independently [37]. Indeed, the smartphone was also programmed to remind the participants about the time each activity was due.

Second, the rather inexpensive (low-cost) technology solution used in this study (i.e., Samsung Galaxy A3 smartphone) provides further support to the idea that everyday technologies can be successfully employed to enable people with disabilities with little or no specific accommodations [38]. Increasingly, researchers as well as educational and rehabilitation professionals are calling for the use of technological solutions that—although not specifically designed for people with disabilities—can be easily ‘repurposed’ to be assistive technologies (e.g., [39]). Moreover, providing accessible and affordable assistive technologies to people with disability is further recognized as a global priority [26]. The system used in this study, which comprises, in addition to a smartphone, an application that can be downloaded free (i.e., Easy Alarm YouTube), does not require any technical skill to be operated, and thus can be easily managed by any professional caregiver without a specific training.

Third, the possibility of having an effective (and affordable) program in support of daily activities of people with intellectual disability or psychiatric disorders is highly relevant for different stakeholders. For the users, for instance, they can reach high levels of independence with personal satisfaction (e.g., increased self-esteem) and with social benefits in terms of image and respect. The positive impact of implementing effective rehabilitation programs supported by assistive/mainstream technologies, however, goes far beyond improving the participation opportunities of individual users [40]. Indeed, there is a well-established link between caregiving for a person with disability and reduced caregivers’ health (e.g., psychological and physical stress) as well as direct and indirect costs such as healthcare, hospital and transportation expenses, caregivers’ loss of working days and earnings, and in general inability for caregivers to maintain a stable employment (for a recent review see [41]). Evidence shows that assistive products can help caregivers by reducing time, levels of assistance and energy put towards caregiving, anxiety and fear, task difficulty, safety risk—particularly for activities requiring physical assistance (e.g., dressing, transferring, toileting, and general mobility)—as well as increasing the independence of the user [42]. With reference to the system used in the present study, one might argue that it can also be relevant for the rehabilitation and care contexts in which the participants operate. In fact, high levels of appropriate initiative (i.e., starting activities at the right time) and performance independence reduces the demand on staff time and efforts, with likely consequences also from an economic standpoint.

### Limitations

Despite the overall positive results, this study has some limitations. First, one may argue that the heterogeneity of the participants’ health conditions (i.e., intellectual disability or psychiatric disorders) may be considered a confounding variable, thus preventing us from drawing definite conclusions about the effectiveness of the program reported as well as its implications. On the other hand, however, such participants’ heterogeneity may be also seen as a strength of this study, in that it may provide evidence of the generalizability of the smartphone-based program to a variety of potential users with different characteristics.

Second, the discrepancy between the high percentage of activities started independently and the relatively frequently low percentage of correct steps for some of the participants calls for further refinement of the program. Specifically, the instructions for the activities were probably vague or delivered at inappropriate times. To address these possible drawbacks, one should probably (a) adjust the instruction expressions so that they fit the participants’ daily jargon and repeat the instructions, (b) allow the use of picture-based instructions to overcome difficulties related to language comprehension, and (c) introduce a basic movement sensor in each activity area so that the smartphone starts providing the step instructions only when the participant has reached such area. Third, no direct assessment was made of the mood of the participants while both interacting with the system and performing the proposed activities. While some anecdotal reports suggested a mood improvement (e.g., smiles) during the intervention sessions, direct evidence is necessary to document the participants’ positive engagement with the program.

Fourth, no social validation was performed to understand the applicability of the proposed smartphone-based program in daily contexts. Future research should involve different stakeholders (e.g., professional caregivers, parents, as well as direct users) to assess their overall attitudes towards the program as well as the barriers and facilitators for its proper implementation in a variety of contexts (e.g., rehabilitation settings, users’ homes).

## 5. Conclusions

The results of this exploratory study suggest that a smartphone-based system can be successfully used to support people with intellectual disability or psychiatric disorders perform a range of functional activities. They also provide evidence of the possibility to enable the participants to initiate such activities on time, without the need to rely on the direct supervision of a caregiver. This study thus provides encouraging evidence on the effectiveness of the proposed system for the person who is willing to receive support from a smartphone when performing functional daily activities as well as his/her context, with potential societal and economic benefits to be further ascertained in future research. Future research might focus on replicating current results involving different participants in other settings (e.g., at home) to assess the generalizability of our results and the applicability of the system to a broader spectrum of disabilities/difficulties, evaluating the wider impact (e.g., social benefits; reduced caregivers’ burden) of this program, assessing the resources needed for its implementation in users’ daily contexts and for longer periods of usage, as well as identifying the most appropriate service delivery approaches to ensure that such solutions are adapted to the specific needs and characteristics of the final users [43].

## Figures and Tables

**Table 1:** Participants’ pseudonyms, chronological age, main diagnosis, and information on intellectual functioning (MMSE; FAB) and (instrumental) activities of daily living (ADL; IADL).

Participant (Pseudonyms)	Chronological Age (Years)	Main Diagnosis	MMSE	FAB	ADL	IADL
Group 1: Psychiatric conditions and cognitive dysfunctions						
Peter (PVA)	50	Schizoaffective disorder	19,89	0	3/6	2/5
Trudy (MR)	48	Major depressive disorder	10,31	0	5/6	7/8
James (RC)	62	Schizophrenia	17,99	0	4/6	4/5
Mary (LA)	47	Schizophrenia	13,62	0	3/6	5/8
Bill (GO)	59	Schizophrenia	22,97	0	2/6	3/5
Anne (DMG)	62	Schizophrenia	22,53	0	3/6	5/8
Jackie (RS)	45	Schizoaffective disorder	22,21	1	2/6	4/8
Ted (VF)	60	Schizophrenia	23,74	0	2/6	4/5
Group 2: Intellectual disability						
Lucas (BG)	40	Intellectual disability *	14,42	0	3/6	4/5
Henry (DLR)	36	Intellectual disability *	22,42	0	2/6	3/5
Jacob (CVG)	25	Severe intellectual disability	15,59	0	3/6	4/5
Sally (NG)	40	Severe intellectual disability	21,42	0	2/6	5/8
Rachel (DMS)	35	Moderate intellectual disability	9,42	0	3/6	5/8
Mason (MG)	27	Severe intellectual disability	15,59	0	2/6	3/5

* Severity of intellectual disability not specified. Abbreviations: MMSE, mini-mental state examination (cut-off: 24); FAB, Frontal Assessment Battery (range min-max: 0–4); ADL, Activities of Daily Living; IADL, Instrumental Activities of Daily Living.

**Table 2:** List of the steps for two of the activities performed.

Exemplar Activity 1: “Brush Your Teeth”	Exemplar Activity 2: “Prepare for Sleep”
1. Go to the bathroom 2. Take your beauty case 3. Open it 4. Take your toothbrush and the toothpaste 5. Take a glass 6. Open the tap 7. Wet your toothbrush 8. Close the tap 9. Put the toothpaste on the toothbrush 10. Start washing your teeth 11. When you are done, rinse your toothbrush 12. Fill the glass and rinse your mouth 13. Throw the glass 14. Put the toothbrush and the toothpaste in the beauty case 15. Wash your hands 16. Dry your hands	Go to your room Take off your shoes Take off your pants Take off your sweatshirt Put them in order in the wardrobe Put on the pajamas Take the keys for the operator Open the locker and take your beauty case Wash your teeth with your toothbrush and the toothpaste Put the beauty case back in its place When you are done, return the keys to the operator Go to your room Go to sleep

## Data Availability

Due to privacy protection agreements, data are not available.
